# Comparison of 90-Day Complication Rates and Cost Between Single and Multiple Joint Procedures for End-Stage Arthropathy in Patients with Hemophilia

**DOI:** 10.2106/JBJS.OA.18.00026

**Published:** 2018-10-23

**Authors:** Bin Feng, Ke Xiao, Peng Gao, Yong Liu, Baozhong Zhang, Yi Ren, Xisheng Weng

**Affiliations:** 1 Department of Orthopaedic Surgery, Peking Union Medical College Hospital, Peking Union Medical College, Beijing, China

## Abstract

**Background:**

Multiple joint procedures during a single anesthetic episode have been proposed for patients with hemophilia as a way to reduce cost. The postoperative 90-day complication rate and the cost distribution between multiple joint procedures and single joint procedures for patients with hemophilia have not been well studied.

**Methods:**

Between January 1996 and December 2016, 124 patients underwent 177 surgical procedures (total knee arthroplasty, total hip arthroplasty, and ankle arthrodesis) for the treatment of hemophilic end-stage arthropathy. Forty-eight patients (39%) underwent multiple joint procedures during 1 hospitalization, and 76 patients (61%) underwent single joint procedures. The medical records were retrospectively reviewed. The patients were evaluated for complications within 90 days postoperatively and the cost during hospitalization. Risk factors related to complications were further analyzed.

**Results:**

Twenty-seven of the 124 patients experienced 29 complications within 90 days postoperatively, representing a complication rate of 16.4% for all procedures. The patients who had undergone multiple joint procedures had a higher rate of surgical complications than those who had undergone a single joint procedure (14.6% vs. 3.9%; p = 0.039). The patients who had had multiple joint procedures had similar rates of hematological complications (8.3% vs. 9.2%; p = 0.867) and total complications (31.3% vs. 18.4%; p = 0.100) compared with those who had had a single joint procedure. There was no difference between the patients who had had multiple joint procedures and those who had had a single joint procedure with regard to the cost of the coagulation factor (p = 0.212).

**Conclusions:**

The performance of multiple joint procedures during a single anesthetic episode is a safe approach for patients with hemophilia with end-stage arthropathy, with no substantial increase in the 90-day complication rate in comparison with that following a single joint procedure. The performance of multiple joint procedures under a single anesthetic episode can save cost and is more cost-effective when managing patients with hemophilia who have end-stage arthropathy.

**Level of Evidence:**

Therapeutic Level III. See Instructions for Authors for a complete description of levels of evidence.

Hemophilia is a hereditary disease that is due to a defect of the X chromosome, which leads to a faulty production of coagulation factor VIII in patients with hemophilia A (85% of cases) and factor IX in patients with hemophilia B. The condition often results in excessive bleeding and leads to musculoskeletal complications^1^. Over 90% of bleeding episodes in patients with hemophilia occur within the musculoskeletal system, increasing the degree of disability and severely affecting the quality of life^2^. Surgical treatment is effective for preserving and restoring function in patients with hemophilia who have end-stage musculoskeletal disorders^3,4^. Because of the natural course of the disease, many patients with hemophilia have multiple joint involvement^5,6^. Multiple joint procedures are defined as any combination of major surgical procedures for the treatment of end-stage arthropathy in patients with hemophilia (including total knee arthroplasty, total hip arthroplasty, and ankle arthrodesis) during a single anesthetic episode. In some studies, simultaneous bilateral total knee arthroplasty has been proposed for patients with hemophilia as a way to reduce costs, improve rehabilitation, and accelerate the return to normal life^7-9^. Multiple joint procedures also can reduce the event of intensive treatment with clotting factor concentrate compared with staged single joint procedures. In patients with hemophilia A, intensive treatment with factor VIII (FVIII) has been associated with FVIII inhibitor development, which is a severe complication that leads to increased postoperative surgery-related and hematological complications and mortality^10^. The literature has also indicated that multiple procedures may be accompanied by increased morbidity and mortality in patients with osteoarthritis^11-13^; however, because limited data are available, it is not known whether the same would be true for patients with hemophilia. The goal of the present study was to evaluate the postoperative 90-day complication rates and the cost distribution between multiple and single joint procedures in patients with hemophilia and end-stage arthropathy.

## Materials and Methods

Following institutional review board approval, the medical records of patients with hemophilia who had undergone primary surgical treatment at our institute between January 1996 and December 2016 were retrospectively reviewed. Patients who had undergone total knee arthroplasty, total hip arthroplasty, and ankle arthrodesis were included. Patients who had undergone revision surgery and those with positive factor inhibitor during the index operation were excluded. A total of 124 patients had undergone 177 surgical procedures (including 106 total knee arthroplasties, 39 total hip arthroplasties, and 32 ankle arthrodeses) for hemophilic end-stage arthropathy at our institution during this period. Demographic data, including age, body mass index (BMI), and type of hemophilia were collected (Table I). Two patients had preoperative human immunodeficiency virus (HIV) infection.

**TABLE I:** Demographic Data*

	SJP Group	MJP Group	P Value†
No. of patients	76	48	
Age‡ *(yr)*	29.1 (19-61)	34.6 (21-56)	0.015
Diagnosis *(no. of patients)*			0.205
Type A	68	46	
Type B	8	2	
BMI‡ *(kg/m*^*2*^*)*	24.2 (17.2-31.4)	25.1 (18.1-28.1)	0.386
Level of clotting factor *(no. of patients)*			0.252
<1%	46	29	
≥1% to ≤5%	20	17	
>5% to ≤25%	9	2	
>25% to ≤40%	1	0	
HIV infection *(no. of patients)*	1	1	
Hepatitis virus infection *(no. of patients)*	16	13	0.44
Length of stay *(d)*	30.1	30.5	0.66

* BMI = body mass index, and HIV = human immunodeficiency virus.

† For continuous variables, an unpaired t test was used. For categorical variables, chi-square analysis was used.

‡ The values are given as the mean, with the range in parentheses.

### Surgical Procedures

All procedures were performed with the patient under general anesthesia. The surgical treatment for patients with hemophilia was patient-specific, and the surgical strategy was decided according to the clinical manifestations and severity of the arthropathy (Table II). The operations were performed by experienced chief orthopaedic surgeons. All total hip arthroplasties were performed through a posterolateral approach with use of cementless implants. All total knee arthroplasties were performed through a midline skin incision and medial parapatellar approach with fixed-bearing posterior-stabilized cemented prostheses. All total knee arthroplasties were performed under tourniquet control, and the synovium was completely removed to reduce recurrent hemarthroses and pain. The ankle arthrodeses were performed with use of intramedullary nailing and either autograft or allograft.

**TABLE II:** Data on Surgical Procedures

		No. of Procedures		
Clinical Manifestation	Surgical Strategy	MJP Group	SJP Group	Total
End-stage knee arthropathy	Total knee arthroplasty	63	43	106
End-stage hip arthropathy	Total hip arthroplasty	27	12	39
End-stage ankle arthropathy	Ankle arthrodesis	11	21	32
Total		101	76	177

Seventy-six patients (61%) underwent single joint procedures (SJP group), and 48 patients (39%) underwent multiple joint procedures during a single anesthetic episode (MJP group) (Table III). The patients in the SJP group had a lower mean age than those in the MJP group; there were no other demographic differences between the groups (Table I).

**TABLE III:** Data on 1-Stage Multiple Joint Procedures for Patients with Hemophilia

Procedure	No. of Patients
3 procedures	
Triple total joint arthroplasty	3
Bilateral total joint arthroplasty + unilateral ankle arthrodesis	2
2 procedures	
Bilateral total joint arthroplasty	36
Total joint arthroplasty + ankle arthrodesis	5
Bilateral ankle arthrodesis	2

### Hematological Care

A preliminary test of factor VIII (FVIII) or factor IX (FIX) was conducted preoperatively for all patients for the purpose of pharmacokinetic evaluation. Plasma-derived FVIII (107 patients) or recombinant FVIII (7 patients) were used for patients with type-A hemophilia, and prothrombin complex concentrates (PCC) were used for patients with type-B hemophilia. The FVIII or FIX level and the factor inhibitor level were regularly monitored. When the inhibitors were detected during the treatment for hemophilia type A, PCC or recombinant coagulation factor VIIa (rFVIIa) was used for the clotting factor replacement therapy.

We referred to the guidelines of World Federation of Hemophilia to assign the strategy of clotting factor replacement therapy^14^. One dose of tranexamic acid was used for patients with type-A hemophilia at the start of the operation. Pharmacological anticoagulation was not used for any patient. Intermittent pneumatic compression was used for mechanical prophylaxis against venous thromboembolism for all patients.

### Clinical Evaluation

All of the patients were evaluated for postoperative complications within 90 days postoperatively. The complications were subdivided into 4 subgroups: surgery-related complications, hematological complications, wound-related complications, and systemic complications. The costs of factor replacement consumption, the total cost of hospitalization, and the cost of implants were further evaluated.

### Statistical Analysis

The clinical data were analyzed with use of mean values. For continuous variables, the unpaired t test was used to assess the difference between the groups; for categorical variables, chi-square analysis was used. The level of significance was set at p < 0.05. To determine the independent predictors associated with postoperative complications, univariate and multivariate logistic regression analyses were performed. Predictor variables for analysis included age (≤20 years, 20 to 40 years, or >40 years), body mass index (BMI) (<20.0, 20.0 to 25.0, or >25.0 kg/m^2^), severity of preoperative clotting factor deficiency (severe or moderate/mild), type of hemophilia (A or B), type of surgery (total knee arthroplasty, total hip arthroplasty, and/or ankle arthrodesis), comorbidity (presence of HIV or hepatitis virus infection), blood transfusion during surgery, and operative strategy (single procedure or multiple procedures). For regression analyses, odds ratios (ORs) and 95% confidence intervals were reported. Significant independent predictor variables were identified as those that maintained a p value of <0.05 and an OR exclusive of 1.0. All statistical analyses were performed with use of SPSS (version 15.0; SPSS).

## Results

All patients were successfully followed for 90 days postoperatively. Twenty-seven patients had 29 complications following 177 total procedures, representing a rate of 16.4% (Table IV). Eleven patients experienced hematological complications (representing a rate of 6.2%), including 6 cases of hematoma, 3 cases of clotting factor inhibitor development, 1 case of deep-vein thrombosis, and 1 case of artery thrombosis. Ten patients had surgery-related complications (representing a rate of 5.6%), including nerve injury, blood vessel injury, deep infection, intraoperative fracture, and failed fusion. Eight patients had wound-related complications (representing a rate of 4.5%), including superficial infection and wound dehiscence. No patient experienced systemic complications. The details of the complications are presented in Table IV.

**TABLE IV:** Summary of Complications After Surgical Treatment for Patients with Hemophilia

	Total Hip Arthroplasty	Total Knee Arthroplasty	Ankle Arthrodesis	Total
No. of procedures	39	106	32	177
Hematological complication				
Hematoma	1	5		6
Inhibitor production	1	2		3
Thrombosis	1	1		2
Wound complication	1	4	3	8
Surgery-related complication				
Nerve injury	1	3		4
Blood vessel injury		1		1
Infection		2	1	3
Fracture	1			1
Failed fusion			1	1
Total	6	18	5	29
Rate per procedure	15.4%	17.0%	15.6%	16.4%

The MJP group had a higher ratio of surgery-related complications and total complications per patient than the SJP group (Table V). Although the difference in surgery-related complications per patient between the MJP and SJP groups was significant, there was no significant difference between the groups in terms of hematological complications, wound complications, postoperative infection, and total complications based on chi-square tests (Table V). Six patients had multiple hospitalizations, with 3 of those 6 patients experiencing postoperative complications. There was a trend toward a higher complication rate among patients with multiple hospitalizations than those with a single hospitalization, but this difference did not reach significance (50% compared with 21%; chi-square = 3.143, p = 0.076). Multivariate logistic regression analysis did not identify significant risk factors for postoperative complications within the selected predictors.

**TABLE V:** Comparison of Postoperative 90-Day Complication Rates per Patient Between SJP and MJP Groups

	SJP Group* (N = 76)	MJP Group* (N = 48)	Chi-Square	P Value
Surgery-related complications	3 (3.9%)	7 (14.6%)	4.489	0.039
Postoperative infection	1 (1.3%)	2 (4.2%)	1.013	0.314
Hematological complications	7 (9.2%)	4 (8.3%)	0.028	0.867
Wound complications	4 (5.3%)	4 (8.3%)	0.459	0.498
Total	14 (18.4%)	15 (31.3%)	2.702	0.100

* The data are given as the number of patients, with the percentage in parentheses.

The mean cost for coagulation factor consumption was $12,300 (United States dollars) for the MJP group and $10,548 for the SJP group (Table VI); this difference was not significant (p = 0.212), which indicated that multiple joint procedures were associated with more cost savings for coagulation factor consumption. The cost of coagulation factor consumption accounted for 47% of the total cost in the MJP group, compared with 57% of the total cost in the SJP group. If each procedure in the MJP group had instead been performed as a single or staged procedure, the estimated cost of the coagulation factor was $22,195 (Table VI), indicating that an additional cost of $9,895 would be charged for the factor consumption.

**TABLE VI:** Cost Comparison

	Cost Comparison			
	SJP Group*	MJP Group*	P Value	Estimated Cost for Staged Multiple Procedures†
Coagulation factor	$10,548	$12,300	0.212	$22,195
Prosthesis	$6,548	$12,214	<0.001	
Total cost	$18,527	$26,096	<0.001	$38,984
Factor cost/total cost	57%	47%		

* The values are given as the average cost after exchange to U.S. dollars.

† The values are given as the average estimated cost if each procedure in the MJP group had instead been performed as staged procedures based on the cost for the SJP group.

## Discussion

Many patients with hemophilia suffer from end-stage arthropathy involving multiple joints^2,5,6,15^. There has been a trend to perform multiple surgical procedures during a single operating session for patients with hemophilia when surgical intervention has been indicated^9,16^. Because a major fraction of the financial burden of surgical treatment for patients with hemophilia is the cost of factor replacement, the benefits of multiple procedures in patients with hemophilia are twofold. First, the costs of the coagulation factor are shared by both procedures and the clotting factor inhibitor formation may be avoided with less clotting factor transfusion. Second, the rehabilitation program is facilitated as a result of the relatively short rehabilitation period following multiple joint procedures as compared with staged procedures^17-19^. Despite the benefits of multiple joint procedures in patients with hemophilia, there have been few reports on multiple procedures in the literature, most of which have mainly focused on arthroplasty and have involved relatively small numbers of cases^7-9^.

We defined multiple joint procedures as those involving some combination of total knee arthroplasty, total hip arthroplasty, and ankle arthrodesis. In the present study, 39% of all patients with hemophilia underwent 1-stage multiple joint procedures. Because of the higher number of procedures and the need for more intensive clotting factor replacement therapy in the MJP group, the consumption of coagulation factor was relatively higher in the MJP group than in the SJP group. Nevertheless, there was not a significant difference in the cost of the coagulation factor between the MJP and SJP groups. The consumption of coagulation factor accounted for 47% of total cost in the MJP group, which was less than that in the SJP group. According to previous studies in the literature, simultaneous bilateral procedures for patients with hemophilia are a cost-effective approach for the treatment of hemophilic arthropathy with equivalent clinical results to unilateral procedures after intermediate-term follow-up^7-9^. The findings in the present study were consistent with those of previous studies^7-9^.

The severe destructive pathological changes associated with arthropathy and the relative osteoporosis associated with hemophilia contribute to the higher surgical complication rate in patients with hemophilia as compared with those without hemophilia^15,20^. In the present study, the complication rate following all procedures was 16.4%. The wound complication rate may be higher in patients with hemophilia^21^ as a result of the deficiency of clot formation after the operation, the poor-quality tissue envelope, and the presence of severe deformity before the operation. The hematological complications were specific for patients with hemophilia and included bleeding or thrombosis secondary to clotting factor replacement and factor inhibitor formation. In light of these findings, surgeons must pay attention during the perioperative treatment of patients with hemophilia and a multidisciplinary team with a hematologist is necessary during the surgical treatment of these patients^20^.

Patients with hemophilia may be presumed to be at low risk for postoperative venous thromboembolism by virtue of their bleeding disorder^22^. There is no consensus about prophylaxis against venous thromboembolism in patients with hemophilia undergoing total joint arthroplasty^22^. Perez Botero et al., in a review of 35 studies on total joint arthroplasty in patients with hemophilia, reported a 0.9% rate of venous thromboembolism following a total of 1,017 total knee and hip arthroplasty procedures and suggested that there is no increased risk for symptomatic venous thromboembolism in patients with hemophilia^23^. The authors concluded that as the incidence of venous thromboembolism in this population is low, joint replacement surgery can be performed safely without routine pharmacological prophylaxis against venous thromboembolism^23^. Such prophylaxis is not used for all patients at our institute. In the present study, in which mechanical prophylaxis was used, the rate of venous thromboembolism after total hip arthroplasty and total knee arthroplasty was 0.7%, which was consistent with the findings of previous studies^23^. It is believed that atherothrombotic events can still occur in patients with hemophilia, although the incidence in such patients is lower than that among patients without hemophilia^24^. Atherothrombotic events associated with the use of PCC for patients with hemophilia is a rare clinical event and has been sporadically reported^25^. The event tends to be associated with large cumulative doses of the coagulation factor^25^. In the present study, 1 patient with type-B hemophilia developed arterial thrombosis after the use of PCC, possibly in relation to the large dose of the factor. Furthermore, atherosclerotic disease and underlying atherosclerotic changes in the patient could not be excluded with certainty.

In the literature, bilateral total knee arthroplasty has been associated with higher perioperative morbidity risk and increased complications than unilateral total knee arthroplasty in patients without hemophilia^26^; however, this scenario may not be true for all patients with hemophilia. In the present study, no patient in the MJP group had systemic complications within 90 days postoperatively. The reason for this finding may have been related to the younger age at the time of the index operation for patients with hemophilia and the rarity of medical comorbidities other than hemophilia^9^.

Previous studies have suggested that simultaneous bilateral total knee arthroplasty and unilateral total knee arthroplasty are associated with similar rates of postoperative complications in patients with hemophilia, leading the authors to conclude that bilateral total knee arthroplasty is a safe and cost-effective approach for such patients^26,27^. The limitation of those studies was the small sample size, which made the results less convincing. In the present study, the MJP group had a significantly higher rate of surgery-related complications; the reason for this finding may have been related to the higher number of procedures in that group. However, the rate of surgery-related complications per procedure was not significantly different between the 2 groups (6.9% in the MJP group compared with 3.9% in the SJP group; p = 0.395), indicating that the performance of multiple joint procedures does not significantly increase the rate of surgery-related complications for each procedure. There also was no significant difference between the MJP and SJP groups in terms of the rate of hematological complications per patient (8.3% compared with 9.2%). In previous reports, more-intensive coagulation factor replacement treatment has been associated with a lower rate of adverse hematological outcomes for patients with hemophilia^28^. This finding may be the reason for the lower rate of hematological complications for the MJP group in the present study^28^. However, the rates of hematological complications, wound complications, and total complications were not significantly different between the 2 groups in the present study. On the basis of the results of the present study, we concluded that multiple joint procedures can be performed safely for patients with hemophilia, without a significant increase in the rates of postoperative complications—including hematological complications—compared with those observed following single procedures.

In the present study, patients who had had multiple hospitalizations had a trend toward a higher complication rate than those who had had a single hospitalization. The reason may have been related to the severity of the disease and more hematological complications^29^. In our study, 3 patients experienced clotting factor inhibitor formation, 2 of whom had multiple hospitalizations. However, the small number of patients with multiple hospitalizations may make the comparison underpowered.

In the present study, multivariate logistic regression analysis did not identify any significant risk factor for postoperative complications in patients with hemophilia. Solimeno et al., in a study of hemophilic patients, previously reported that the presence of coagulation factor inhibitor and a high HIV-positive rate were correlated with a higher rate of postoperative complications^30^. The present study did not demonstrate such correlations. The reason may be related to the lower rate of HIV infection and the small number of patients with clotting factor inhibitor formation in the present study. Again, the small number of HIV-positive patients in the present study may make the conclusion underpowered.

The present study had several limitations. First, this was a single-center retrospective study from China, which may not be reflective of other centers. Second, because hemophilia is a rare disease and the present study was retrospective, no estimation of the necessary sample size was performed prior to the study. The study may not have been adequately powered to discover the difference in complications between multiple and single joint procedures. An attempt at post-hoc power calculation for surgery-related complications per procedure estimated that at least 270 patients would have been needed in order to demonstrate a significant difference. Further prospective well-designed studies with sufficient numbers of patients are needed. Third, the present study evaluated complications that occurred within 90 days postoperatively in order to increase the likelihood that the complications were mainly related to the hemophilia and perioperative management. Thus, late complications outside of this window, such as periprosthetic joint infection and loosening, were not evaluated in the present study. Additional studies with long-term follow-up are needed to assess long-term complications as well as quality of life. Last, we only studied the total cost and the cost of the coagulation factor; we did not consider other hospitalization fees and the indirect costs of treatment such as the days off work and outpatient rehabilitation.

In conclusion, although multiple joint procedures were associated with a higher rate of surgery-related complications per patient than single joint procedures in the present study, the multiple and single joint procedures were associated with similar rates of hematological and total complications. Our findings suggest that multiple joint procedures can provide cost savings and are more cost-effective for the treatment of end-stage arthropathy in patients with hemophilia.
